# Neurocognitive Dysfunction in Fibrosing Interstitial Lung Diseases: A Multidimensional Analysis of Pulmonary, Cognitive, and Clinical Correlates

**DOI:** 10.3390/diagnostics16010004

**Published:** 2025-12-19

**Authors:** Zsolt Vastag, Emanuela Tudorache, Daniel Traila, Ioana Ciortea, Ovidiu Fira-Mladinescu, Cristian Oancea, Felix Bratosin, Elena Cecilia Rosca

**Affiliations:** 1 Center for Research and Innovation in Personalised Medicine of Respiratory Diseases (CRIPMRD), Pulmonology University Clinic, “Victor Babes” University of Medicine and Pharmacy, 300041 Timisoara, Romania; zsolt.vastag@umft.ro (Z.V.); ioana.ciortea@umft.ro (I.C.); mladinescu@umft.ro (O.F.-M.); oancea@umft.ro (C.O.); 2 Department of Internal Medicine, Discipline of Clinical Practical Skills, “Victor Babes” University of Medicine and Pharmacy, 300041 Timisoara, Romania; 3 Doctoral School, “Victor Babes” University of Medicine and Pharmacy, 300041 Timisoara, Romania; 4 Pulmonology Department, “Victor Babes” University of Medicine and Pharmacy, 300041 Timisoara, Romania; 5 Department of Biology and Life Sciences, “Vasile Goldis” University, 310002 Arad, Romania; 6 Department of Infectious Diseases, “Victor Babes” University of Medicine and Pharmacy, 300041 Timisoara, Romania; felix.bratosin@umft.ro; 7 Department of Neurology, “Victor Babes” University of Medicine and Pharmacy, 300041 Timisoara, Romania; rosca.elena@umft.ro; 8 Department of Neurology, Clinical Emergency County Hospital Timisoara, 300736 Timisoara, Romania

**Keywords:** interstitial lung diseases, cognition disorders, pulmonary function tests, neuropsychological tests, spirometry

## Abstract

**Background and Objectives:** Fibrosing interstitial lung diseases (ILDs) may predispose to neurocognitive impairment through chronic hypoxemia and systemic inflammation, yet data integrating pulmonary physiology, disease severity, and cognition are limited. We aimed to compare global cognitive performance between adults with fibrosing ILD and contemporaneous non-ILD clinic comparators, explore differences across ILD subtypes, and identify physiologic and clinical predictors of low MMSE scores. **Materials and Methods:** In this single-center cross-sectional study, 45 adults with fibrosing ILD and 32 non-ILD participants from university-affiliated pulmonology clinics completed the Mini-Mental State Examination (MMSE) and standardized lung function testing (including diffusing capacity, DLCO%). Comorbidity (Charlson index), inflammatory markers (C-reactive protein), and GAP (Gender–Age–Physiology) severity were recorded. Associations with MMSE and MMSE < 24 were examined using correlations and multivariable logistic regression. **Results:** Mean MMSE was lower in ILD than in non-ILD participants (23.9 ± 3.6 vs. 26.8 ± 2.8; *p* < 0.001), and MMSE < 24 occurred in 33.3% versus 12.5%, respectively. Within ILD, the usual interstitial pneumonia (UIP) pattern showed the lowest MMSE scores. DLCO% and total lung capacity correlated positively with MMSE (r = 0.44 and r = 0.34, respectively). In multivariable models, ILD diagnosis remained associated with MMSE < 24 (odds ratio [OR] 2.72, 95% CI 1.14–6.48), and each 10-percentage-point decrement in DLCO% increased the odds of MMSE < 24 (OR 1.42, 95% CI 1.11–1.92). GAP ≥ 4 was also associated with impaired cognition (OR 2.91, 95% CI 1.13–7.57). **Conclusions:** Fibrosing ILD, particularly with reduced diffusing capacity and higher GAP stage, is associated with lower MMSE scores and a higher frequency of values below a conventional impairment threshold. Prospective studies incorporating comprehensive neuropsychological testing are needed to determine whether and how neurocognitive assessment should be integrated into routine ILD care.

## 1. Introduction

Idiopathic pulmonary fibrosis (IPF) remains the prototypical fibrosing interstitial lung disease (ILD), with a usual interstitial pneumonia (UIP) pattern on high-resolution computed tomography (HRCT) and histopathology. Contemporary guidelines emphasize multidisciplinary diagnosis and increasingly recognize the construct of progressive pulmonary fibrosis (PPF) across non-IPF ILDs, underscoring shared pathobiology and trajectories of decline [1,2].

Beyond respiratory morbidity, chronic lung disease has been linked to neurocognitive dysfunction through convergent mechanisms that include sustained hypoxemia, systemic inflammation, oxidative stress, and microvascular injury. Reviews synthesize evidence for structural and functional brain changes in chronic respiratory disease—ranging from white-matter abnormalities to deficits in attention, executive function, and memory—suggesting a lung–brain axis that may accelerate cognitive aging [3,4].

Epidemiologic and imaging studies increasingly connect reduced pulmonary function to cerebral small-vessel disease (CSVD)—a major substrate for vascular cognitive impairment. In population cohorts, lower spirometric indices correlate with magnetic resonance imaging (MRI)-defined lacunes and white-matter lesions, independent of smoking, supporting a vascular pathway by which impaired pulmonary physiology may compromise cerebral perfusion and oxygen delivery [5]. Authoritative reviews of Cerebral Small Vessel Disease (CSVD) detail how endothelial dysfunction, hypoperfusion, and inflammatory cascades drive cognitive decline, motor slowing, and gait instability, providing a biologically plausible framework for pulmonary-to-cerebral signaling [6].

Although cognitive impairment has been extensively characterized in obstructive lung diseases, ILD-specific data are emergent. Early case–control work in IPF demonstrated lower performance across attention, processing speed, and learning domains compared with healthy controls, particularly in those with advanced diffusion impairment [7]. Subsequent studies across idiopathic interstitial pneumonia (IIP) phenotypes corroborated decrements in multiple domains—often in association with reduced DLCO and exercise desaturation—yet sample sizes were modest and instruments varied [8]. A recent scoping review highlighted the paucity of high-level evidence, identified DLCO, hypoxemia, and disease phenotype (e.g., IPF/UIP) among the factors most consistently linked to worse cognition, and called for multidimensional analyses integrating pulmonary physiology, disease severity indices, and comorbidities [9].

Sleep-disordered breathing (SDB) represents a further, potentially modifiable contributor to neurocognitive risk in ILD. Obstructive sleep apnea (OSA) is highly prevalent in IPF—prospective and retrospective series and a recent meta-analysis estimate that roughly two-thirds of patients have OSA—and has been associated with poorer neurocognitive performance and lower nocturnal oxygen saturations [10,11,12]. These data suggest that intermittent hypoxemia and sleep fragmentation may compound daytime cognitive deficits in fibrosing ILDs and strengthen the rationale for routine screening and targeted management of SDB within comprehensive ILD care pathways [10,11,12].

Risk-stratification frameworks such as the GAP (Gender–Age–Physiology) and ILD-GAP models capture survival risk across IPF and non-IPF ILDs and can be augmented by comorbidity indices to refine prognosis [13,14,15]. Yet whether these validated staging tools parallel neurocognitive vulnerability—and how they interact with physiologic gas-exchange metrics, systemic inflammatory markers, and multimorbidity—remains insufficiently defined. Accordingly, we hypothesize that ILD is independently associated with worse global cognitive performance relative to non-ILD comparators, and that greater restriction and gas-exchange impairment (particularly lower DLCO), higher disease-severity stage (GAP/ILD-GAP), and higher systemic inflammatory burden will be associated with lower cognitive scores. Our objective is to provide a multidimensional assessment of pulmonary, cognitive, and clinical correlates to clarify which physiologic and clinical factors most robustly predict neurocognitive dysfunction in fibrosing ILD. According to the European Respiratory Society (ERS)/American Thoracic Society (ATS) 2025 update on multidisciplinary classification of the Interstitial Pneumonias, fibrotic ILDs are divided based on radiological–pathological criteria into UIP, NSIP, Bronchiolocentric interstitial pneumonia (BIP), Diffuse alveolar damage (DAD), Pleuroparenchymal fibroelastosis (PPFE), Lymphoid interstitial pneumonia (LIP), combined pattern, and unclassifiable pattern [16].

## 2. Materials and Methods

### 2.1. Study Design and Setting

We conducted a single-center, observational cross-sectional study at the ‘Victor Babeș’ University of Medicine and Pharmacy Timișoara, Romania, using patients evaluated in the university-affiliated pulmonology clinics. Participants were consecutively enrolled between October 2022 and September 2025, after ethics approval and until the planned sample size was reached. Recruitment and assessments were coordinated through the university’s respiratory research units and conducted in the same academic clinical environment described in prior institutional work, with standardized procedures harmonized across clinics and laboratories. The study period encompassed consecutive enrollments until the planned sample size was attained.

PICO statement: The study population consisted of adults evaluated in university pulmonology clinics, including patients with fibrotic ILD [classified by multidisciplinary discussion (MDD) into IPF, connective-tissue disease-associated fibrotic ILD, hypersensitivity pneumonitis (HP), idiopathic nonspecific interstitial pneumonia (iNSIP), sarcoidosis-associated pulmonary fibrosis (SAPF), and unclassifiable ILD] and a contemporaneous non-ILD comparator group. The “intervention/exposure” was the presence of fibrosing ILD (and, secondarily, ILD subtype and GAP severity), contrasted with non-ILD participants as the comparator. The primary outcome was global cognitive performance (MMSE total score, with secondary analyses of MMSE domains and MMSE < 24). The overarching objective was to quantify differences in cognition between ILD and non-ILD groups, to explore cognitive heterogeneity across ILD subtypes, and to identify clinical and physiologic predictors—particularly diffusing capacity (DLCO%) and GAP severity—associated with low cognitive performance.

### 2.2. Legal and Ethical Considerations

The protocol was reviewed and approved by the Local Commission of Ethics for Scientific Research in accordance with Romanian Law no. 95/2006, Order 904/2006, the EU Good Clinical Practice Directive 2005/28/EC, ICH–GCP, and the Declaration of Helsinki. All participants provided written informed consent prior to any study procedure. Data were pseudonymized at source, stored on secure institutional servers, and analyzed only in aggregate form; no identifiable information was retained in the analytic datasets.

### 2.3. Inclusion and Exclusion Criteria

Eligible participants were adults (≥18 years) able to complete neurocognitive testing in Romanian and to undergo standardized lung function testing within the same evaluative visit. The fibrosing interstitial lung disease (ILD) group comprised patients with a multidisciplinary diagnosis of ILD established according to contemporaneous ATS/ERS guidance (integrating clinical evaluation, HRCT patterning, respiratory functional testing, serology, and depending on the case, bronchoalveolar lavage or lung biopsy) [16]. ILD subtypes recorded a priori were UIP, NSIP, BIP, sarcoidosis-associated pulmonary fibrosis, and unclassifiable/mixed pattern. The comparator group included adults without clinical or radiologic evidence of interstitial disease, evaluated contemporaneously in the same university-affiliated pulmonology clinics for non-fibrotic respiratory complaints (e.g., chronic cough, suspected obstructive airway disease, or unexplained dyspnea) and meeting the same eligibility criteria. Although all comparators were free of ILD, their specific non-fibrotic diagnoses were not retained as a structured variable in the analytic dataset.

Exclusion criteria applicable to both groups were any acute exacerbation of respiratory disease within four weeks; acute or chronic hypoxemia, decompensated cardiac, renal, or hepatic failure precluding testing; current delirium or acute confusional state; prior neurological disorders known to impair global cognition (dementia, major stroke with residual aphasia); uncorrected visual or hearing deficits interfering with testing; and inability to complete the Mini-Mental State Examination (MMSE). For analytic consistency, participants with incomplete primary outcome data (MMSE total score) or key pulmonary function indices (DLCO%, total lung capacity—TLC%) were excluded.

### 2.4. Procedures and Measurements

All evaluations were completed during a single visit whenever feasible. Trained clinicians administered the MMSE in Romanian under standardized, distraction-free conditions. In addition to the total MMSE score (0–30), we recorded domain subscores (orientation, registration, attention/calculation, recall, and language) using the canonical scoring rubric. Pulmonary function was assessed in accordance with ATS/ERS standards. Spirometry provided Forced Vital Capacity (FVC) and Forced Expiratory Volume in 1 s (FEV1), and the FEV1/FVC ratio was calculated from best acceptable maneuvers. Body plethysmography yielded TLC% and residual volume (RV%). Single-breath diffusing capacity for carbon monoxide (DLCO%) and transfer coefficient (KCO%) were measured with correction for the participant’s hemoglobin; percent predicted values were derived from reference equations implemented by the institutional laboratory. Oxygen saturation at rest on room air was documented.

Peripheral venous blood was sampled on the same day for routine biomarkers, including C-reactive protein (CRP), erythrocyte sedimentation rate (ESR), hemoglobin, differential leukocyte count (eosinophils), urea, and creatinine. Assays were performed in the hospital’s ISO-certified core laboratory using validated methods with ongoing internal quality control. Comorbidity burden was quantified by the Charlson Comorbidity Index (CCI). Among ILD participants, disease severity was indexed with the GAP index using sex, age, FVC%, and DLCO%; GAP stage and a dichotomized severity threshold (GAP ≥ 4 vs. < 4) were pre-specified. Smoking status (never, former, current) was ascertained by structured interview and chart verification.

### 2.5. Study Variables and Outcomes

The primary outcome was global cognitive performance by MMSE analyzed as a continuous score and, in secondary analyses, as a binary endpoint using the customary impairment threshold MMSE < 24 [17]. Key pulmonary predictors were DLCO%, KCO%, TLC%, RV%, FVC%, and the FEV1/FVC ratio. Inflammatory and hematologic biomarkers (CRP, ESR, eosinophils, hemoglobin, urea, creatinine) were secondary predictors. The principal exposure was diagnostic group (fibrotic ILD vs. non-ILD). Within the ILD group, exploratory prespecified subgroup analyses contrasted UIP, NSIP, BIP, sarcoidosis-associated pulmonary fibrosis and unclassifiable ILD. Additional exploratory analyses examined GAP severity (GAP ≥ 4) in relation to cognitive outcomes.

We did not systematically collect data on educational attainment, detailed sleep metrics, mood symptoms, or psychotropic medication use in a structured format suitable for analysis; these potentially important determinants of cognitive performance are therefore not included as covariates in the present models.

### 2.6. Definitions

ILD subtypes followed multidisciplinary discussion, integrating clinical–radiologic–pathologic data; morphological pattern was defined on HRCT and histology (where needed). Restrictive ventilatory impairment was inferred from reduced TLC% with preserved or elevated FEV1/FVC ratio; gas-exchange impairment was indexed by reduced DLCO% and KCO%. Low cognitive performance was defined as MMSE < 24, recognizing that this threshold indicates at least mild global impairment in clinical and research settings. CRP and ESR were interpreted as systemic inflammation markers; eosinophils were analyzed as a percentage of leukocytes. GAP staging categorized severity as Stage I–III and was additionally dichotomized at ≥4 points to denote more advanced disease.

### 2.7. Sample Size and Power Considerations

Before enrollment, we targeted a medium standardized difference in MMSE between ILD and non-ILD groups (Cohen’s d ≈ 0.6), which under α = 0.05 and power = 0.80 yields a minimum total sample of nearly 72 participants for two-group comparisons; to accommodate for potential missingness and ensure adequate precision for subgroup and multivariable analyses, we planned approximately 75–80 participants. The final analytic sample comprised 77 individuals (45 ILD and 32 non-ILD), aligning with the original target and ensuring ≥80% power to detect clinically meaningful between-group MMSE differences, given observed variances.

### 2.8. Statistical Analysis

Continuous variables are reported as mean ± standard deviation (SD) if normally distributed, or median with interquartile range (IQR) if skewed. Categorical variables are shown as frequencies or percentages. Between-group comparisons (ILD vs. non-ILD) used independent-samples *t*-tests or Mann–Whitney U tests, as appropriate. One-way ANOVA or Kruskal–Wallis tests evaluated differences among three or more subgroups (e.g., UIP, NSIP, other). When overall tests were significant, we conducted post hoc pairwise comparisons via Bonferroni correction to control for type I error. We performed Pearson’s or Spearman’s correlations (depending on normality) to assess relationships between MMSE scores and numeric parameters (DLCO%, CRP, etc.). Multiple logistic regression was applied to identify independent predictors of MMSE < 24, with age, sex, ILD diagnosis, DLCO%, CRP, and CCI as covariates. Continuous predictors were entered as continuous terms without categorization; DLCO% was scaled per 10-percentage-point decrement and CRP per 5 mg/L increase to aid interpretability of odds ratios. The dependent variable in these models was binary (MMSE < 24 vs. ≥ 24. Statistical significance was set at two-sided *p* < 0.05.

## 3. Results

Table 1 offers a comparative overview of demographic characteristics and key comorbidities in the ILD (*n* = 45) and non-ILD (*n* = 32) cohorts. The mean age is slightly higher in ILD at 65.2 years, yet this did not reach statistical significance (*p* = 0.19). A near-equal proportion of men appears in both groups (58% vs. 59%), indicating no major sex imbalance. The prevalence of smoking history—known to influence both pulmonary function and extra-pulmonary outcomes—was somewhat greater in ILD (42% vs. 34%), though not significantly (*p* = 0.43). Comorbidity burdens, reflected by the CCI, are modestly higher in ILD (3.7 ± 2.1 vs. 3.1 ± 1.9), but again no statistical difference emerges (*p* = 0.25). Hypertension (HBP) and diabetes rates are also similar across the two populations, suggesting that common cardiometabolic conditions are comparably distributed. Dyslipidemia was more frequently recorded among ILD participants (26.7% vs. 18.8%), yet the difference is not conclusive (*p* = 0.42). Non-ILD participants were primarily evaluated for non-fibrotic respiratory complaints (such as chronic cough, suspected obstructive airway disease, or unexplained dyspnea) and had no clinical or radiologic evidence of interstitial lung disease, but their specific non-fibrotic diagnoses were heterogeneous and not tabulated as a separate analytic variable.

Table 2 illustrates the ILD cohort stratified by subtype, focusing on markers of disease severity. The GAP index (Gender, Age, Physiology) median values show moderate severity overall. The UIP subgroup (n = 15) exhibits the highest GAP index median at 4 (IQR 3–5), suggesting greater disease burden than NSIP (3 [2,3,4]) or sarcoidosis-associated pulmonary fibrosis (2 [1,2,3]). A one-way ANOVA comparing FVC% among these subgroups yielded an overall *p* < 0.05, indicating that at least one pairwise contrast is significant. Post hoc Bonferroni analysis reveals that UIP patients have significantly lower FVC% (66.2 ± 14.5) than NSIP patients (72.3 ± 12.1, *p* = 0.041), consistent with UIP being the more fibrotic and functionally limiting phenotype. FEV1% tends to mirror FVC% patterns, with UIP showing the lowest mean (68.7 ± 13.2) and sarcoidosis registering the highest mean (78.3 ± 9.1), although not statistically significant.

Both DLCO (46.8 ± 15.7 vs. 71.4 ± 18.2) and KCO are lower in the ILD group compared with the non-ILD group (68.5 ± 15.2 vs. 86.9 ± 14.3, *p* = 0.002), reinforcing that alveolar membrane pathology affects gas-transfer efficiency. Total lung capacity (TLC%) shows a classic restrictive pattern in ILD (67.3 ± 12.0) relative to non-ILD (83.6 ± 14.2, *p* < 0.001). Residual volume (RV%) is also diminished in ILD, reflecting reduced lung volumes overall (*p* = 0.007). Notably, FVC% (forced vital capacity) is significantly compromised in ILD (69.7 ± 13.4 vs. 82.4 ± 14.5, *p* = 0.001), though the FEV1/FVC ratio remains normal or even slightly elevated (81.6 ± 8.2 vs. 78.9 ± 7.5, *p* = 0.13). This is typical for restrictive diseases, wherein both FEV1 and FVC decrease proportionally (Table 3).

**Table 3:** Pulmonary function test comparison: ILD vs. non-ILD.

Parameter	ILD (*n* = 45)	Non-ILD (n = 32)	*p*-Value
DLCO%	46.8 ± 15.7	71.4 ± 18.2	<0.001
KCO%	68.5 ± 15.2	86.9 ± 14.3	0.002
TLC%	67.3 ± 12.0	83.6 ± 14.2	<0.001
RV%	59.1 ± 18.1	71.9 ± 19.7	0.007
FVC%	69.7 ± 13.4	82.4 ± 14.5	0.001
FEV1/FVC Ratio	81.6 ± 8.2	78.9 ± 7.5	0.13

Values are mean ± SD unless indicated. DLCO% = diffusing capacity of the lung for carbon monoxide, percent predicted; KCO% = transfer coefficient (DLCO/alveolar volume), percent predicted; TLC% = total lung capacity, percent predicted; RV% = residual volume, percent predicted; FVC% = forced vital capacity, percent predicted; FEV1/FVC = ratio of forced expiratory volume in one second to forced vital capacity. Comparisons are ILD vs. non-ILD with two-sided tests (α = 0.05).

Table 4 presents cognitive performance, measured by MMSE, stratified by broad diagnostic categories (ILD vs. non-ILD) and further broken down by ILD subtypes. A striking difference emerges: the mean MMSE for the ILD group is 23.9 ± 3.6, significantly lower than 26.8 ± 2.8 in the non-ILD cohort (*p* < 0.001 by *t*-test), and nearly one-third (33.3%) of ILD patients fall below the <24 cutoff consistent with at least mild cognitive impairment. Among ILD subtypes, those with the UIP pattern show the lowest average MMSE (22.8 ± 3.5) and the highest frequency (46.7%) of scores <24. By contrast, NSIP patients exhibit a somewhat higher mean MMSE (24.9 ± 3.2) and fewer individuals below the cutoff (25%). The one-way ANOVA across ILD subtypes is significant (*p* = 0.02), and Bonferroni post hoc testing indicates a significant difference between UIP and NSIP (*p* = 0.04). Other fibrotic ILD patterns (e.g., Bronchiolocentric interstitial pneumonia) cluster closer to NSIP in cognitive scores. The small sarcoidosis-associated pulmonary fibrosis subgroup (n = 3) had numerically higher mean MMSE scores (25.7), but this subgroup is too small for reliable inference, and these values should be interpreted as purely descriptive.

Table 5 presents individual MMSE domains, providing insight into which cognitive aspects are disproportionately impacted in ILD versus non-ILD. Orientation scores are marginally lower in ILD (9.3 vs. 9.7, *p* = 0.05), hinting at early deficits in time and place awareness. Registration (i.e., immediate memory) also shows a mild but significant decline (2.3 vs. 2.7, *p* = 0.02). Strikingly, the largest differences lie in attention/calculation (2.9 ± 1.4 in ILD vs. 4.2 ± 1.0 in non-ILD, *p* < 0.001) and recall (1.4 ± 0.9 vs. 2.2 ± 0.8, *p* < 0.001). Language scores, however, do not differ significantly (7.9 vs. 8.0, *p* = 0.62).

Table 6 compares laboratory parameters between ILD and non-ILD participants, offering potential insights into systemic inflammation, oxygen transport capacity, and metabolic status. Hemoglobin levels average around 13.9 g/dL in ILD, slightly lower but not statistically different from the non-ILD mean of 14.3 g/dL. Eosinophil percentages appear higher in ILD (2.4 vs. 1.9), yet this did not reach significance (*p* = 0.16), suggesting that eosinophilic inflammation is not a universal driver of ILD (though it can be relevant in specific entities such as eosinophilic pneumonia). CRP and ESR, broad markers of inflammation, trend higher in ILD (14.2 vs. 8.5 mg/L and 19.7 vs. 13.5 mm/h, respectively), with borderline significance (CRP *p* = 0.06, ESR *p* = 0.07).

Table 7 underscores the relationships between cognitive performance (MMSE) and various physiologic or inflammatory markers. Pearson’s correlation coefficients demonstrate a moderate positive link between MMSE and DLCO% (r = 0.44, *p* = 0.0005), KCO% (r = 0.37, *p* = 0.002), TLC% (r = 0.34, *p* = 0.006), and FVC% (r = 0.31, *p* = 0.01). In essence, better pulmonary function—particularly higher diffusing capacity (DLCO, KCO)—is associated with better cognitive scores. These findings bolster the hypothesis that alveolar gas-exchange capacity may influence cerebral oxygenation and thus cognitive processes.

The analysis also presents a post hoc evaluation comparing individuals with MMSE < 24 to those scoring ≥24, illustrating more severe physiologic compromise in the cognitively impaired group. For instance, their mean DLCO% is 38.4 versus 52.1 (*p* = 0.002), representing a substantial decrement in diffusing capacity. Similar patterns emerge for KCO% (62.1 vs. 72.4, *p* = 0.01), TLC% (61.0 vs. 72.7, *p* = 0.008), and FVC% (64.2 vs. 72.3, *p* = 0.03). CRP, while weakly negatively correlated (r = −0.22, *p* = 0.07), does show a trend toward higher values in those with lower MMSE (17.5 vs. 11.1 mg/L, *p* = 0.09), but the difference lacks strict significance.

**Figure 1:**
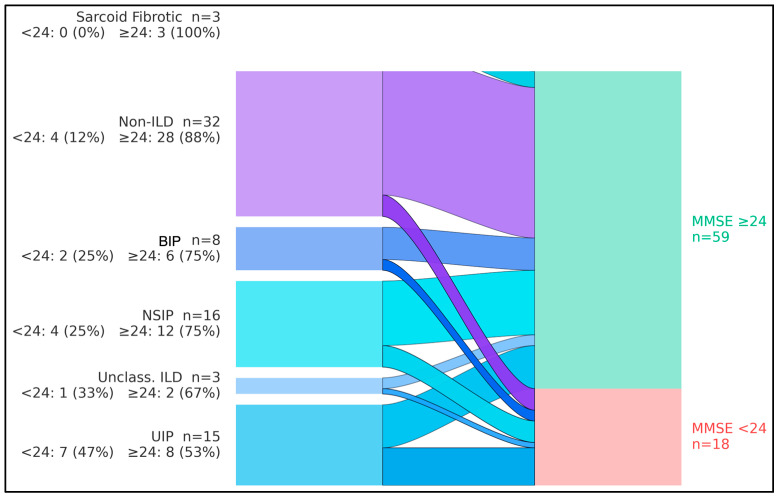
Flow of diagnostic groups into cognitive status categories. Flow diagram (alluvial layout) illustrating the distribution of Mini-Mental State Examination (MMSE) scores ≥ 24 versus < 24 across fibrosing interstitial lung disease (ILD) subtypes and non-ILD comparators. Bars on the left represent diagnostic groups [usual interstitial pneumonia (UIP), nonspecific interstitial pneumonia (NSIP), bronchiolocentric interstitial pneumonia (BIP), sarcoid-fibrotic disease, unclassifiable/combined-pattern ILD, and non-ILD], whereas bars on the right represent MMSE categories (MMSE ≥ 24, MMSE < 24). The figure highlights that the UIP pattern contributes a disproportionately large share of individuals with MMSE < 24 compared with other ILD subtypes and non-ILD participants.

The flows showed that, overall, 18/77 participants (23.4%) had MMSE < 24 and 59/77 (76.6%) had MMSE ≥ 24. By diagnostic group, cognitive impairment (MMSE < 24) occurred in 7/15 with UIP (46.7%), 4/16 with NSIP (25.0%), 2/8 with BIP pattern (25.0%), 1/3 with unclassifiable/combined pattern ILD (33.3%), 0/3 with sarcoidosis-associated pulmonary fibrosis (0%), and 4/32 among non-ILD comparators (12.5%). Consequently, UIP contributed the largest share of impaired cases (7/18, 38.9%), whereas non-ILD participants predominantly exhibited preserved cognition (28/32, 87.5%). The aggregate streams converged to a larger node for MMSE ≥ 24 (n = 59) and a smaller node for MMSE < 24 (n = 18), indicating that most groups retained normal cognition while impairment clustered in fibrotic ILD—particularly UIP (Figure 1).

Table 8 presents a multivariate logistic regression model identifying predictors of cognitive impairment (MMSE < 24) across the entire sample (n = 77). Even after adjusting for age, comorbidity burden (CCI), DLCO%, and CRP, having an ILD diagnosis is significantly associated with increased odds of cognitive impairment (OR = 2.72, 95% CI 1.14–6.48, *p* = 0.024). This finding highlights that ILD status itself confers an independent contribution to poorer cognitive outcomes.

Among continuous predictors, each 10% decrement in DLCO% elevates the risk of low MMSE by approximately 42% (OR = 1.42, *p* = 0.008), emphasizing the importance of gas-exchange capacity. CRP was not a statistically significant predictor in this model (*p* = 0.10), and any apparent association should be considered exploratory; these data do not support a strong independent effect of CRP on MMSE < 24 once pulmonary physiology and comorbidity are taken into account. Age, expressed per five-year increment, shows a borderline effect (*p* = 0.09), reflecting the general principle that advancing age can modestly increase the odds of cognitive decline.

We also included GAP ≥ 4 as a dichotomous variable for ILD participants, representing more advanced disease. ILD patients with GAP ≥ 4 have nearly triple the odds of impaired cognition (OR = 2.91, *p* = 0.026) compared to those with lower GAP scores, underscoring that disease severity is a key driver. Overall, this model supports the notion that ILD severity and reduced DLCO% are robust predictors of cognitive risk, reinforcing calls for integrated pulmonary and neurocognitive assessment in patients with fibrotic lung disease.

**Figure 2:**
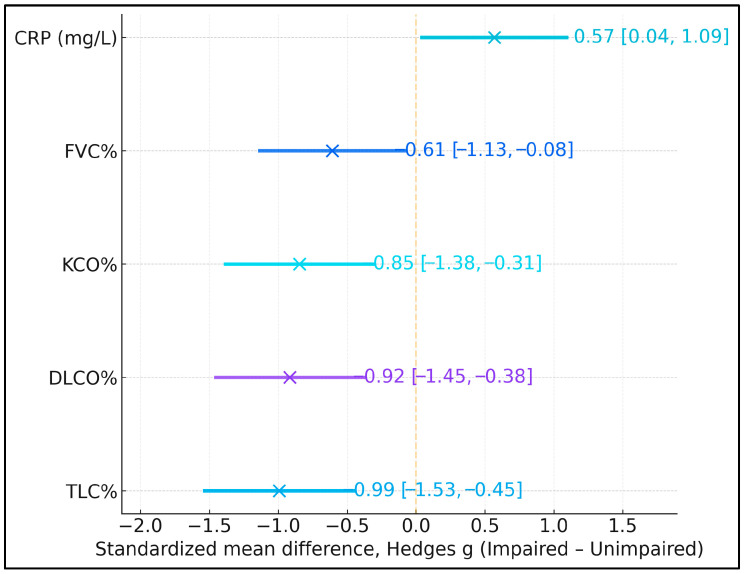
Standardized mean differences (Hedges g) in lung volumes, gas-transfer capacity, and C-reactive protein (CRP) between participants with Mini-Mental State Examination (MMSE) < 24 and ≥24. Negative values indicate lower mean values in the cognitively impaired group (MMSE < 24), whereas positive values indicate higher mean values. Error bars represent 95% confidence intervals.

Standardized mean differences (Hedges g; impaired minus unimpaired) indicated that the cognitively impaired group had markedly lower lung volumes and gas-transfer capacity, alongside somewhat higher systemic inflammation. Effect sizes for TLC%, DLCO%, KCO%, and FVC% ranged from −0.61 to −0.99, corresponding to moderate-to-large deficits in pulmonary function among participants with MMSE < 24 (Figure 2). In contrast, C-reactive protein (CRP) showed a moderate positive effect size (g = 0.57, 95% CI 0.04 to 1.09), indicating higher inflammatory burden in the impaired group, although this finding should be interpreted cautiously and in conjunction with regression results, where CRP did not independently predict MMSE < 24.

## 4. Discussion

### 4.1. Analysis of Findings

The present analysis demonstrated that adults with fibrotic ILD had lower global cognitive performance than contemporaneous non-ILD clinic comparators, with approximately one-third of ILD participants scoring below a conventional MMSE impairment threshold and the most pronounced deficits observed in the UIP pattern. Within MMSE domains, attention/calculation and recall were most affected, suggesting that fronto-subcortical networks supporting working memory and attentional control may be particularly vulnerable in this population. These findings are consistent with prior case–control data in idiopathic interstitial pneumonia and mild IPF showing lower MMSE and neuropsychological scores relative to healthy controls [18,19], and they extend this literature by demonstrating graded relationships between diffusion impairment, GAP severity, and MMSE-defined cognitive status.

Notably, domains reliant on attention and recall were disproportionately affected in the current cohort, although the use of a single MMSE administration precludes firm conclusions about stable domain-specific impairment versus fluctuating attentional performance. Collectively, convergent evidence supported a clinically meaningful association between fibrotic ILD phenotypes—especially UIP—and reduced performance on global and domain-specific cognitive measures [18,19].

Physiological correlates in this study—particularly the graded relationship between lower DLCO/TLC and lower MMSE and the independent contribution of ILD status after adjustment—were coherent with population-based neuroimaging and aging-cohort findings linking impaired pulmonary function to brain structural injury and subsequent cognitive decline. In community samples, reduced forced vital capacity was associated with heavier cerebral small-vessel disease burdens (white-matter hyperintensities, lacunes), providing a plausible vascular substrate for cognitive impairment when gas-exchange capacity was compromised [20]. Longitudinal data in older adults further suggested that lower FEV1/FVC predicted incident cognitive decline and dementia-related outcomes, reinforcing the concept that deteriorating lung function tracked with cognitive aging trajectories [21]. In parallel, systems-level work implicated inflammatory and endothelial pathways as mechanistic bridges between pulmonary dysfunction and microvascular brain injury, complementing the current observation that CRP showed only a weak, inconsistent association with MMSE when modeled alongside physiology [22].

In order to eliminate a confounder, we excluded patients with acute or chronic respiratory failure from our cohort. However, mechanistic physiological studies using near-infrared spectroscopy during exercise offered additional granularity regarding the link between gas exchange and cognition-relevant brain physiology. Patients with IPF who desaturated during exertion exhibited an early and disproportionate fall in cerebral oxygenation, even at low workloads, compared with non-desaturators; correcting desaturation attenuated these cerebral oxygenation abnormalities [23]. In ILD, targeted oxygen supplementation during constant-load cycling improved prefrontal cortical oxygenation and reduced perceived fatigue, directly tying reversal of cerebral hypoxia to a patient-centered outcome [24]. These studies could at least partially explain our impairment findings in several domains (attention/calculation, recall) by suggesting that intermittent cerebral hypoxemia during daily exertion could preferentially burden frontal–subcortical networks subserving attention and working memory.

Translational implications followed from these converging physiologic and mechanistic signals. A recent meta-analysis of randomized trials in fibrotic ILD concluded that supplemental oxygen improved exercise SpO_2_, endurance, dyspnea, and fatigue, with trends favoring high-flow systems; although long-term cognitive effects were not evaluated, the demonstrated improvements in exertional hypoxemia and fatigue provided a rationale for testing cognition as an endpoint in future trials [25]. Contemporary rehabilitation frameworks for ILD emphasized the intertwined nature of physical and cognitive function and advocated combining exercise training with cognitive-supportive strategies (e.g., pacing, dual-task training, cueing) to translate physiologic gains into meaningful daily activities—an approach that aligned with the present finding that disease severity (GAP ≥ 4) tracked with cognitive risk [26]. On this basis, routine cognitive screening, preferential attention to patients with markedly reduced DLCO, and integration of oxygen titration during training appeared justified as pragmatic steps within comprehensive ILD care.

Subtype-specific signals in the current cohort also warranted comment. The sarcoidosis-associated pulmonary fibrosis subgroup was very small (n = 3), and thus any apparent preservation of MMSE scores must be regarded as exploratory and non-inferential. Nonetheless, the absence of marked MMSE deficits in these three participants is not inconsistent with prior objective testing that failed to detect frank executive dysfunction in sarcoidosis-absent neurosarcoidosis [27]. At the same time, larger cohort analyses demonstrated that subjective cognitive difficulties were common in sarcoidosis and independently associated with poorer health-related quality of life, and prospective data implicated everyday cognitive failures as a determinant of fatigue—highlighting that patient-reported cognitive burden may exceed what brief global screens capture [28,29]. These nuances reinforced the need to (i) consider diagnosis-specific mechanisms (e.g., neurosarcoidosis, sleep disturbance, mood/fatigue) when interpreting cognitive measures and (ii) complement MMSE with targeted domain testing and patient-reported outcomes, particularly in non-UIP ILDs where overt deficits may be subtle or context-dependent [27,28,29]. Nevertheless, these findings may be influenced by patient-specific, environmental, healthcare-related, and study design factors; therefore, our results should be interpreted within the appropriate context [30,31,32,33,34,35,36].

The findings supported integrating routine neurocognitive assessment into fibrotic ILD care, with priority given to patients showing greater physiologic restriction, diffusion impairment, or advanced stage. Because cognitive performance tracked gas-exchange and lung-volume limitations, management reasonably emphasized optimization of oxygenation at rest and on exertion, early and progressive pulmonary rehabilitation with cognitive-supportive strategies, and systematic evaluation for modifiable contributors such as sleep-disordered breathing and medication effects. Subtype signals indicating greater vulnerability in UIP, together with severity gradients, justified risk-stratified workflows that included timely neuropsychology referral, caregiver education, and incorporation of cognitive status into shared decision-making about antifibrotic therapy, rehabilitation goals, safety counseling, and advance care planning.

### 4.2. Study Limitations

This cross-sectional analysis from an academic center limited causal inference and generalizability, and small diagnostic subgroups constrained precision for between-phenotype contrasts. Second, cognitive status was assessed using a single global screening tool (MMSE) administered at one time point. MMSE performance is sensitive to transient influences such as attention, engagement, fatigue, and testing context, and cannot, by itself, distinguish stable neurocognitive impairment from more isolated attentional fluctuations. Moreover, the MMSE has limited sensitivity for subtle executive and visuospatial deficits compared with instruments such as the Montreal Cognitive Assessment (MoCA). Our findings should therefore be interpreted as preliminary and hypothesis-generating; future ILD studies should incorporate MoCA and detailed neuropsychological batteries to better delineate domain-specific profiles and confirm the robustness of the observed associations. Third, while all non-ILD comparators had no clinical or radiologic evidence of fibrosing interstitial disease, their underlying non-fibrotic respiratory diagnoses were heterogeneous and not stratified in our analyses, so we cannot exclude residual confounding from conditions that themselves may affect cognition. Reliance on a brief global screen introduced ceiling and floor effects and offered limited domain specificity, while the absence of comprehensive neuropsychological testing or neuroimaging precluded the localization of deficits. Residual confounding likely persisted despite adjustment. In particular, we did not capture educational attainment, detailed sleep measures (polysomnography, validated sleep questionnaires), mood or anxiety symptoms, or psychotropic medication use in a structured way, and these unmeasured factors may have influenced MMSE scores and contributed to the observed differences between groups. Nocturnal oximetry was not systematically captured, biomarker profiling was restricted to routine inflammatory indices, and multiple exploratory comparisons increased the possibility of spurious findings. Longitudinal trajectories and responses to targeted interventions were not assessed.

## 5. Conclusions

ILD was associated with clinically meaningful decrements in global cognition, most evident in fibrotic phenotypes such as UIP and in patients with more advanced disease, and cognitive performance paralleled the severity of restriction and gas-exchange impairment. These observations support the concept of a lung–brain axis in fibrotic ILD and suggest that consideration of risk-stratified cognitive assessment, alongside oxygenation-focused, rehabilitative, and sleep-targeted interventions, may be appropriate within comprehensive care. However, given the cross-sectional design and reliance on a single global screener, these findings should be viewed as hypothesis-generating and are insufficient on their own to mandate routine cognitive screening in all patients with ILD. Future multicenter longitudinal studies incorporating detailed neuropsychological profiling and mechanistic biomarkers are warranted to clarify causality, delineate diagnosis-specific patterns, and identify modifiable targets to preserve cognitive health in ILD.

## Figures and Tables

**Table 1:** Baseline demographics and key comorbidities.

Variable	ILD (*n* = 45)	Non-ILD (n = 32)	*p*-Value
Age (years), mean (SD)	65.2 ± 8.4	62.7 ± 9.1	0.19
Male Sex, n (%)	26 (57.8)	19 (59.4)	0.88
Smoking History, n (%)	19 (42.2)	11 (34.4)	0.43
CCI, mean (SD)	3.7 ± 2.1	3.1 ± 1.9	0.25
HBP, n (%)	26 (57.8)	17 (53.1)	0.69
Diabetes, n (%)	9 (20.0)	6 (18.8)	0.88
Dyslipidemia, n (%)	12 (26.7)	6 (18.8)	0.42

Data are presented as mean ± standard deviation (SD) or n (%). ILD = interstitial lung disease; non-ILD = without interstitial lung disease; CCI = Charlson Comorbidity Index; HBP = high blood pressure; n = number of participants. Two-sided *p*-values correspond to between-group tests specified in Methods; α = 0.05; Non-ILD participants were evaluated for non-fibrotic respiratory complaints in the same pulmonology clinics and had no clinical or radiologic evidence of ILD; specific non-fibrotic diagnoses were heterogeneous and not stratified in this table.

**Table 2:** Disease severity indices within the ILD group.

ILD Subtype Pattern	*n*	GAP Index, Median (IQR)	FVC% (Mean ± SD)	FEV1% (Mean ± SD)	*p*-Value (ANOVA)
UIP	15	4 (3–5)	66.2 ± 14.5	68.7 ± 13.2	>0.05
NSIP	16	3 (2–4)	72.3 ± 12.1	74.9 ± 14.3	<0.05
BIP	8	3 (2–5)	70.0 ± 11.3	72.5 ± 10.8	>0.05
Sarcoidosis	3	2 (1–3)	76.5 ± 9.0	78.3 ± 9.1	>0.05
Unclass./Comb. ILD	3	2 (1–4)	68.1 ± 11.7	71.2 ± 12.5	>0.05

Data are shown as median (interquartile range, IQR) for GAP and mean ± SD for spirometry. ILD subtypes: UIP = usual interstitial pneumonia; NSIP = nonspecific interstitial pneumonia; BIP = Bronchiolocentric interstitial pneumonia; Unclass./Comb. ILD = unclassifiable/combined pattern ILD.

**Table 4:** Cognitive outcomes (MMSE) by diagnostic group and ILD subtype.

Group/Subgroup	*n*	Mean MMSE (± SD)	MMSE < 24, *n* (%)	*p*-Value (ANOVA)
Non-ILD (All)	32	26.8 ± 2.8	4 (12.5)	>0.05
ILD (All)	45	23.9 ± 3.6	15 (33.3)	<0.001 * (vs. Non)
UIP	15	22.8 ± 3.5	7 (46.7)	>0.05
NSIP	16	24.9 ± 3.2	4 (25.0)	0.02 **
BIP	8	24.6 ± 3.1	2 (25.0)	>0.05
SAPF	3	25.7 ± 2.1	0 (0.0)	>0.05
Unclass./Comb. Pattern ILD	3	24.5 ± 3.0	1 (33.3)	>0.05

Data are mean ± SD or n (%). MMSE = Mini-Mental State Examination. ILD subtypes: UIP = usual interstitial pneumonia; NSIP = nonspecific interstitial pneumonia; BIP = Bronchiolocentric interstitial pneumonia; SAPF = sarcoidosis-associated pulmonary fibrosis; Unclass./Comb pattern ILD = unclassifiable/combined pattern ILD. Groupwise test among ILD subtypes used one-way ANOVA with Bonferroni-adjusted post hoc comparisons. Single asterisk indicates ILD vs. non-ILD *t*-test (* *p* < 0.001). Double asterisk indicates overall ANOVA significance among ILD subtypes (** *p* = 0.02); reported post hoc *p*-values are Bonferroni-adjusted. Bonferroni post hoc: UIP vs. NSIP *p* = 0.04.

**Table 5:** Sub-analysis: MMSE domain scores in ILD vs. non-ILD.

Domain	ILD (Mean ± SD)	Non-ILD (Mean ± SD)	*p*-Value
Orientation (0–10)	9.3 ± 1.1	9.7 ± 0.8	0.05
Registration (0–3)	2.3 ± 0.8	2.7 ± 0.6	0.02
Attention/Calculation (0–5)	2.9 ± 1.4	4.2 ± 1.0	<0.001
Recall (0–3)	1.4 ± 0.9	2.2 ± 0.8	<0.001
Language (0–9)	7.9 ± 1.6	8.0 ± 1.2	0.62

Domain scores are reported as mean ± SD. MMSE domains: orientation (0–10), registration (0–3), attention/calculation (0–5), recall (0–3), language (0–9). MMSE = Mini-Mental State Examination. Between-group *p*-values are two-sided with α = 0.05.

**Table 6:** Laboratory variables compared between ILD vs. non-ILD.

Marker	ILD (*n* = 45)	Non-ILD (*n* = 32)	*p*-Value
Hemoglobin (g/dL)	13.9 ± 1.4	14.3 ± 1.5	0.24
Eosinophils (%)	2.4 ± 1.5	1.9 ± 1.2	0.16
CRP (mg/L)	14.2 ± 12.1	8.5 ± 7.0	0.06
ESR (mm/h)	19.7 ± 14.8	13.5 ± 10.4	0.07
Urea (mg/dL)	35.9 ± 15.1	31.3 ± 13.7	0.21
Creatinine (mg/dL)	0.90 ± 0.25	0.88 ± 0.22	0.65

Values are mean ± SD. CRP = C-reactive protein (mg/L); ESR = erythrocyte sedimentation rate (mm/h). ILD = interstitial lung disease. Hemoglobin is in g/dL; urea and creatinine are in mg/dL. Two-sided *p*-values correspond to tests specified in Methods; α = 0.05.

**Table 7:** Correlation and post hoc analysis: MMSE vs. pulmonary function.

Parameter	Pearson’s r (MMSE)	*p*-Value	Post Hoc Analysis (MMSE < 24 vs. ≥24)
DLCO%	0.44	0.0005	38.4 ± 14.2 vs. 52.1 ± 15.0 (*p* = 0.002)
KCO%	0.37	0.002	62.1 ± 11.9 vs. 72.4 ± 12.1 (*p* = 0.01)
TLC%	0.34	0.006	61.0 ± 9.8 vs. 72.7 ± 12.2 (*p* = 0.008)
FVC%	0.31	0.01	64.2 ± 11.5 vs. 72.3 ± 13.7 (*p* = 0.03)
CRP (mg/L)	−0.22	0.07	17.5 ± 13.8 vs. 11.1 ± 10.2 (*p* = 0.09)

Correlations use Pearson’s r unless otherwise specified; r = correlation coefficient. MMSE = Mini-Mental State Examination; DLCO% = diffusing capacity of the lung for carbon monoxide, percent predicted; KCO% = transfer coefficient (DLCO/alveolar volume), percent predicted; TLC% = total lung capacity, percent predicted; FVC% = forced vital capacity, percent predicted; CRP = C-reactive protein. Post hoc comparisons contrast MMSE < 24 vs. ≥ 24 with two-sided tests and α = 0.05.

**Table 8:** Multivariate logistic regression for MMSE < 24.

Predictor	Odds Ratio (95% CI)	*p*-Value
ILD Diagnosis	2.72 (1.14–6.48)	0.024
Age (per 5 years)	1.18 (0.97–1.42)	0.09
CCI (per unit)	1.10 (0.90–1.34)	0.34
DLCO% (per 10% ↓)	1.42 (1.11–1.92)	0.008
CRP (per 5 mg/L)	1.04 (0.99–1.09)	0.1
GAP ≥ 4 (vs. <4)	2.91 (1.13–7.57)	0.026

Multivariable logistic regression models for the odds of MMSE < 24. OR = odds ratio; CI = confidence interval; CCI = Charlson Comorbidity Index; DLCO% = diffusing capacity of the lung for carbon monoxide, percent predicted; CRP = C-reactive protein; GAP = Gender–Age–Physiology index. “Per 10% ↓” denotes the effect per 10-percentage-point decrement in DLCO%. Covariates and model specification are detailed in Methods; two-sided α = 0.05.

## Data Availability

The datasets generated during and/or analyzed during the current study are not publicly available due to privacy (GDPR) restrictions. However, anonymized datasets may be available upon reasonable request and with appropriate ethical approval. Requests to access the datasets should be directed to Dr. Zsolt Vastag (zsolt.vastag@umft.ro).
